# Regional differences in the global burden of age-related macular degeneration

**DOI:** 10.1186/s12889-020-8445-y

**Published:** 2020-03-30

**Authors:** Xiayan Xu, Jing Wu, Xiaoning Yu, Yelei Tang, Xiajing Tang, Xingchao Shentu

**Affiliations:** Department of Ophthalmology, The Second Affiliated Hospital of Zhejiang University, College of Medicine, Zhejiang, Hangzhou China

**Keywords:** Macular degeneration, Disability-adjusted life years, Global burden of disease

## Abstract

**Background:**

Age-related Macular Degeneration (AMD) is the leading cause of blindness. This study aims to analyze regional differences on the global burden of AMD and help direct related policy making.

**Methods:**

Disability-adjusted life years (DALY) data were collected from the Global Burden of Disease Study (GBD) 2017 to estimate the AMD burden. Mean education years, human development index (HDI) and Public Health Expenditure were extracted from the Human Development Report 2018, and latitude data were obtained from the Google Earth. These four factors were analyzed to see their importance in regional differences of AMD burden, using Kruskal-Wallis test, Dunn’s multiple comparisons test as well as regression analysis.

**Results:**

Global age-standardized DALY rates have decreased since 2011. Based on the WHO region system, age-standardized DALY rates in African and Eastern Mediterranean region were significantly lower than those of other four regions. Linear regression analysis indicated that age-standardized DALY rates were inversely related to HDI and mean education years.

**Conclusions:**

The age-standardized AMD burden had a decreasing tendency recently. Lower socioeconomic status and fewer education years were associated with higher AMD burden. The finding of this study may highlight the importance of national development and education on relieving AMD burden.

## Background

Age-related Macular Degeneration (AMD) is a progressive disease that preferentially affects the macular region of the retina [1]. It has a total population of about 170 million individuals who are affected with AMD globally, thus being the third leading cause of vision loss in the world [2, 3]. In the United States, the prevalence of AMD reaches 11 million, similar to that of all invasive cancers combined, leading to an annual $4.6 billion direct healthcare cost [4]. However, different regions showed different patterns of AMD burden. The Beijing Eye Study [5, 6] showed that visual loss owing to AMD was comparatively uncommon in China, and obviously less than in the Western. On the contrary, some other studies [7, 8] presented that the prevalence of AMD in Asian populations was comparable with white populations. In the Singapore Chinese Eye Study [9], it was reported that early AMD was more common in Chinese and Indians than in Malays, further underlining the widespread difference in regions. To better explain this difference, we use disability-adjusted life years (DALYs) as a main measurement to make comparisons across latitude, socioeconomic status, education years, public health expenditure and so on. DALYs are the sum of years lived with disability (YLDs) and years of life lost (YLLs) owing to premature death. As for AMD, DALYs equal YLDs in general. Therefore, this study was to explore the underlying mechanisms on the difference of AMD burden among different regions, using the DALY data available from the Global Burden of Disease (GBD) 2017 study [10].

## Methods

### Overview

The GBD study aims to explore health loss due to diseases, injuries, and risk factors by age, sex, and geography. Additional detail on GBD metrics and definitions can be found elsewhere [11]. Detailed methods for estimating DALYs have been mentioned in related publications [12, 13]. Importantly, all countries share the same disability weights [14]. There were no ethical issues involved in this study as the data shown here were all extracted from the websites online.

### Data extraction

Global burden of AMD was estimated in terms of DALYs in the GBD 2017 study including 189 countries. The following data regarding AMD burden were collected [10]: (1) global DALYs, DALYs per 100,000 population and age-standardized DALYs per 100,000 population during 1990 to 2015, (2) national age-standardized DALYs per 100,000 population in 2015 and 2017.

Google Earth was used to identify the latitude of each country, with < 30° defined as low latitude, > 60°as high latitude, and as medium latitude when in between.

The Human Development Index (HDI) is a composite measurement of health, education and income of a country, ranging from 0 to 1, with a higher value representing a corresponding higher level of socioeconomic status. Based on the Human Development Report [15], the data were extracted from the United Nations Development Programme (UNDP) database as follows: (1) HDI data in 2017, (2) national mean education years in 2017 and (3) national public health expenditure in 2015. According to the UNDP categorization, countries were divided into 4 groups in 2017: very high, high, medium and low.

### Statistical analysis

The differences of age-standardized DALY rates among three latitude-based country groups as well as four HDI-based groups were compared using Kruskal-Wallis test, followed by Dunn’s multiple comparisons test [16, 17]. Linear regression analysis was performed to investigate the effect of national HDI, national mean education years and national public health expenditure on age-standardized DALY rates. All statistical analyses were performed using SPSS 24 (IBM Corporation, Chicago, Illinois, USA). *P* values of less than 0.05 were considered statistically significant.

## Results

### AMD burden by year

**Fig. 1:**
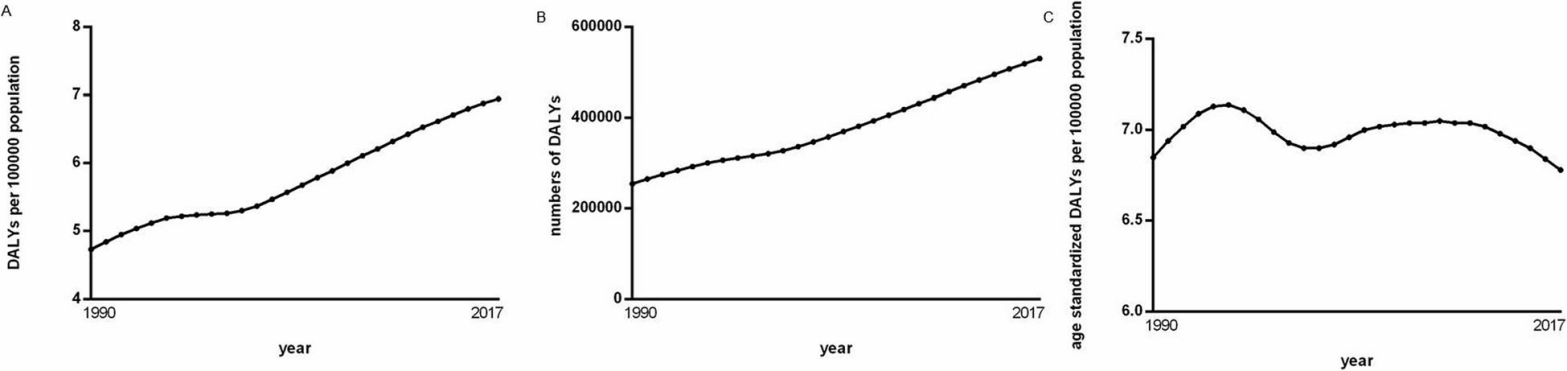
Changes in global burden of Age-related Macular Degeneration (AMD) in terms of disability-adjusted life years (DALY) numbers (left), DALY rates (middle), and age-standardized DALY rates (right) between 1990 and 2017. Lines represent 95% uncertainty intervals

Global DALY numbers caused by AMD rose by 108.2%, from 2.5 million in 1990 to 5.3 million in 2017 (Fig. 1a). Consistently, DALY rates increased by 46.9% from 4.73 to 6.95 after controlling population size between 1990 and 2017 (Fig. 1b). On this basis, we further removed the impact of a growing world population and composition changes during the same period, showing that the AMD burden in terms of age-standardized DALY rates increased slightly by 4.2% from 6.85 to 7.14 during 1990 to 1995, then dropped by 3.4% to 6.9 in 2000, followed by a relatively constant period until 2011, and decreased year by year during 2011 to 2017 (Fig. 1c).

### AMD burden by region in 2017

**Fig. 2:**
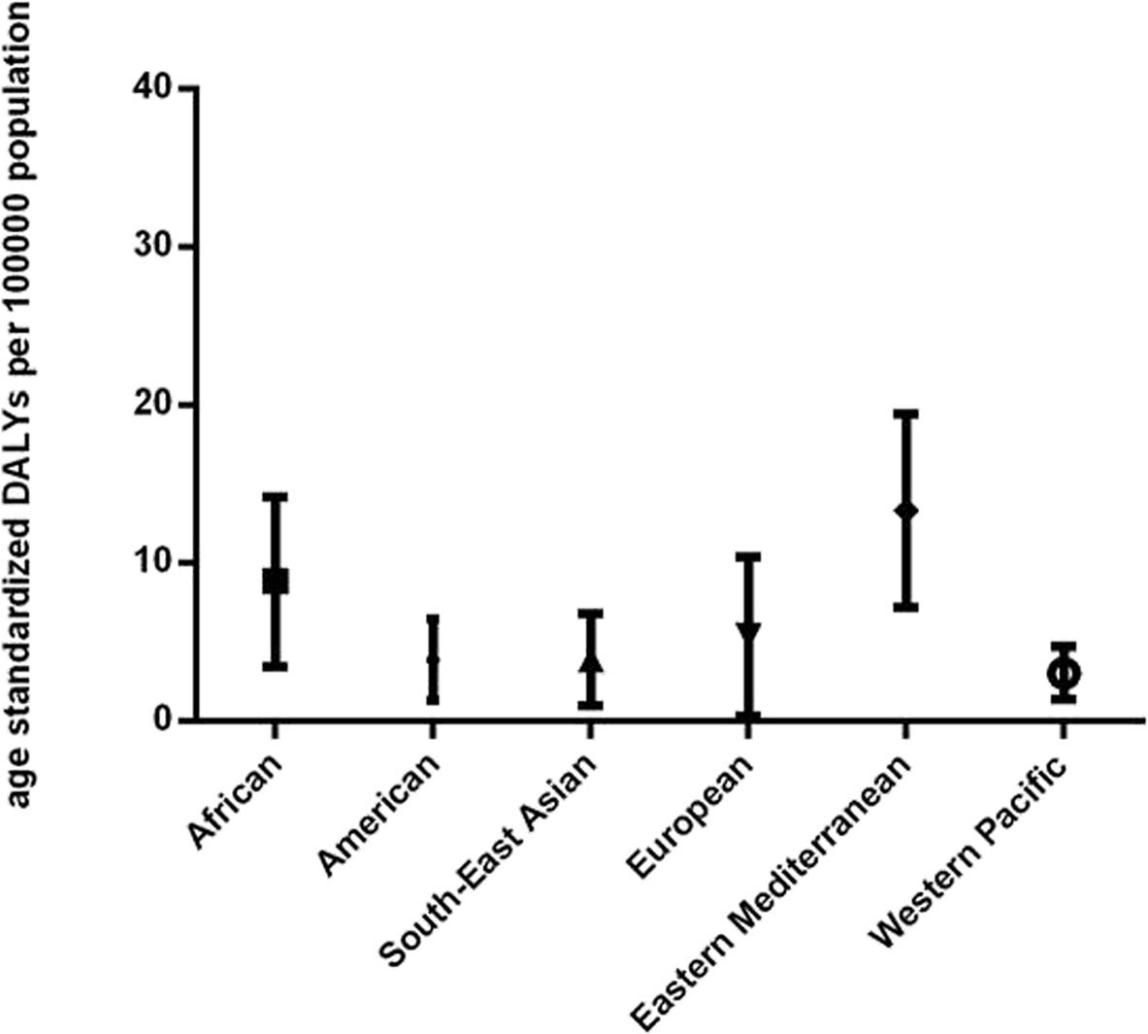
Global burden of Age-related Macular Degeneration (AMD) in terms of age-standardized disability-adjusted life years (DALY) rates by World Health Organization (WHO) region in 2017. Lines represent 95% uncertainty intervals

There are 189 countries in total included in the GBD 2017 study, covering 47 African countries, 35 American countries, 10 South-East Asian countries, 50 European countries, 21 Eastern Mediterranean countries and 24 Western Pacific countries. Results of analysis using Kruskal-Wallis test showed a significant difference of age-standardized DALYs per 100,000 population among six WHO regions (*P* < 0.0001, Fig. 2). Further performing Dunn’s multiple comparison test showed that the AMD burdens of African and Eastern Mediterranean regions were significantly higher than American, South-East Asian, European and Western Pacific regions (*P* = 0.0004, *P* = 0.0421, *P* = 0.0002, *P* < 0.0001, respectively in African comparisons; P < 0.0001, *P* = 0.0003, P < 0.0001, P < 0.0001, respectively in Eastern Mediterranean comparisons).

### AMD burden by latitude in 2017

**Fig. 3:**
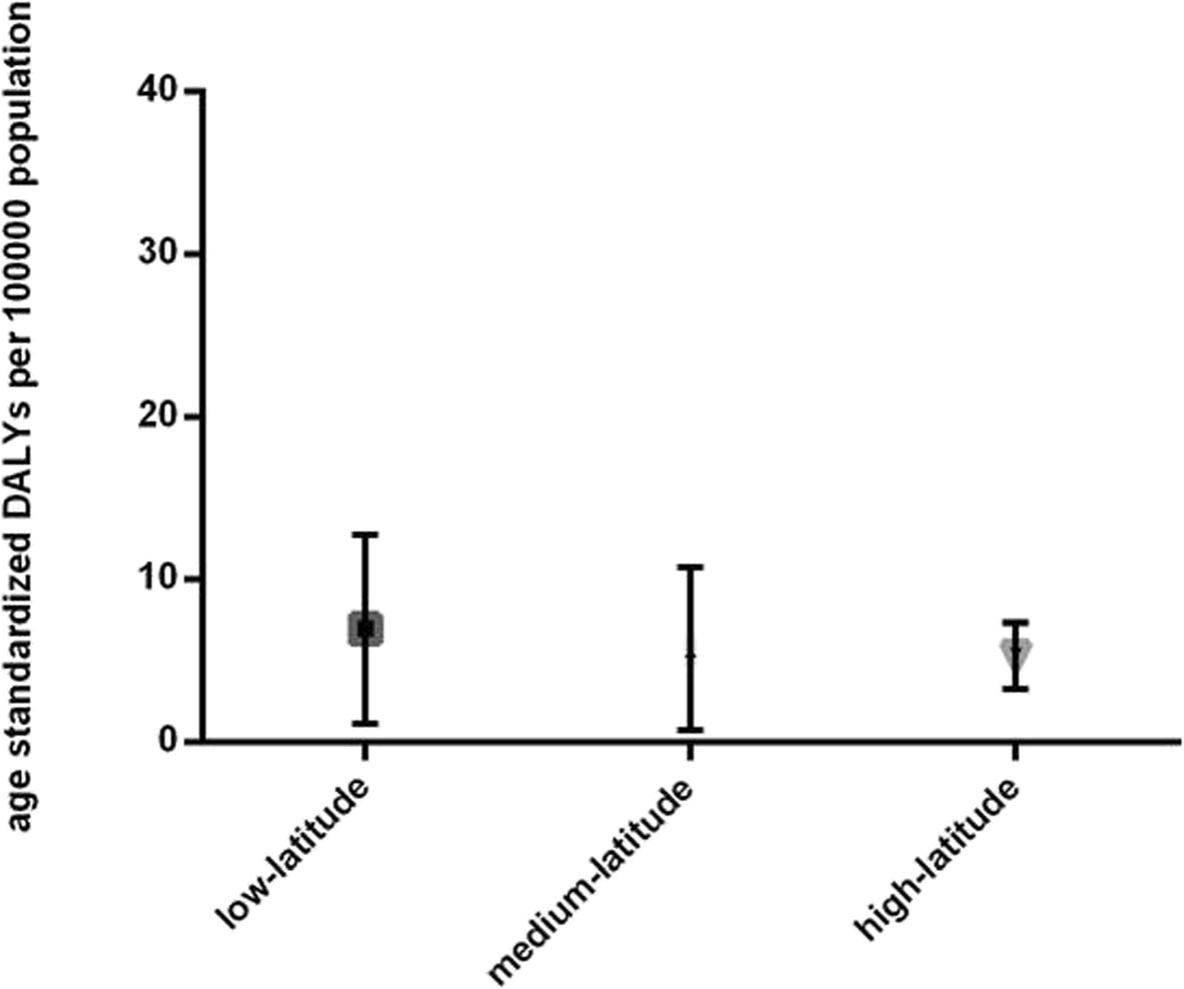
Global burden of Age-related Macular Degeneration (AMD) in terms of age-standardized disability-adjusted life years (DALY) rates by national latitude in 2017. AMD burden differed among countries with different latitude levels. Lines represent 95% uncertainty intervals

Latitude data were available for 189 countries by Google Earth, including 4 high-latitude countries, 67 medium-latitude countries and 118 low-latitude countries. Kruskal-Wallis test indicated that age-standardized DALY rates shared no significant difference among countries of different latitude groups (*P* = 0.0896, Fig. 3).

### AMD burden by socioeconomic development in 2017

**Fig. 4:**
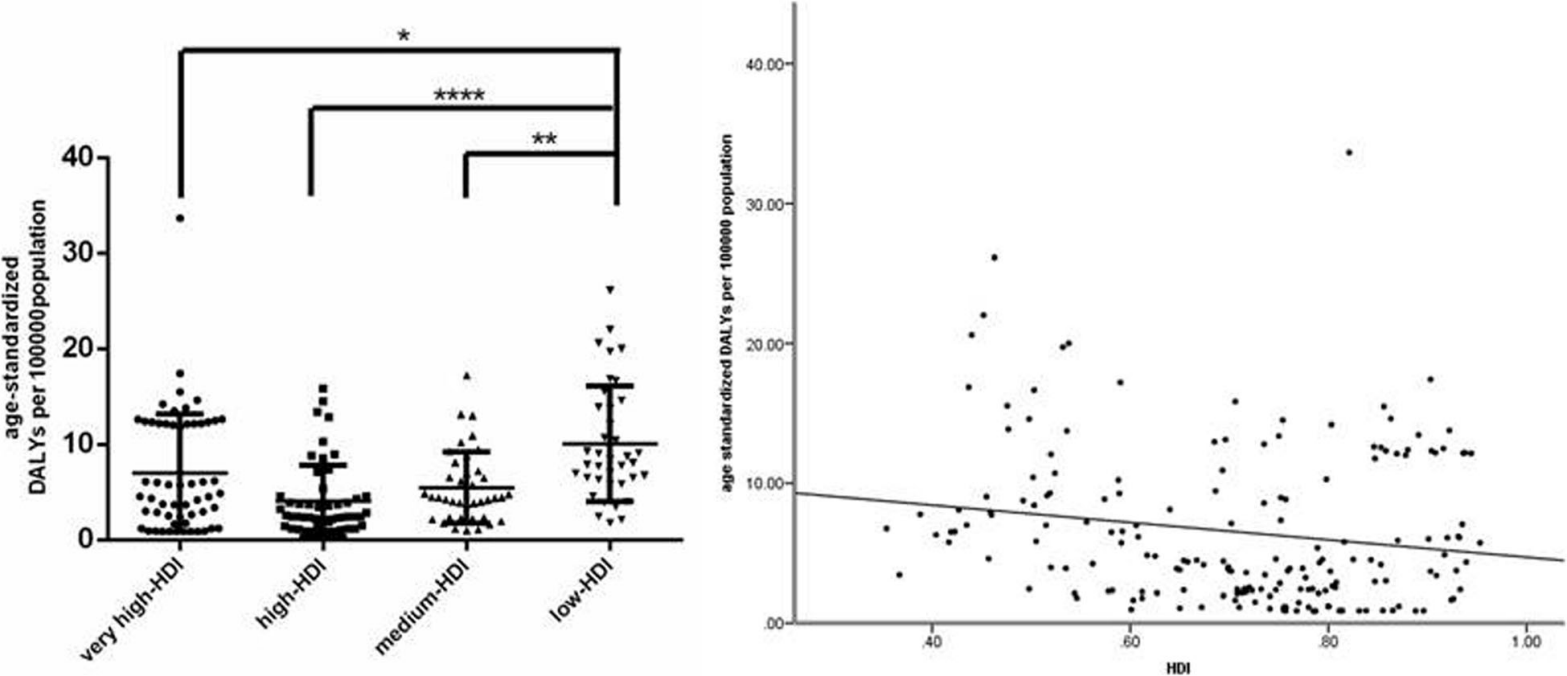
Global burden of Age-related Macular Degeneration (AMD) in terms of age-standardized disability-adjusted life years (DALY) rates by national socioeconomic development in 2017. (Left) AMD burden differed among countries with different socioeconomic development levels. Lines represent the median burden. *P < 0.05, ***P < 0.001, ****P < 0.0001. (Right) AMD burden was inversely associated with socioeconomic development level. The line represents a linear fit

HDI data in 2017 were available for 189 countries, including 59 very high-HDI, 53 high-HDI, 39 medium-HDI and 38 low-HDI countries. Kruskal-Wallis tests indicated that age-standardized DALY rates differed significantly among countries with different socioeconomic development levels (*P* < 0.0001). Multiple comparisons using Dunn’s test revealed that very high-, high- and medium- HDI countries had significantly higher age-standardized DALY rates than low-HDI ones (Fig. 4a). Linear regression analysis suggested that HDI had a significant effect on age-standardized DALY rates (adjusted R^2^ = 0.024; *P* = 0.019). As can be seen in Fig. 4b, age-standardized DALY rates were inversely associated with HDI (standardized β = − 0.173, P = 0.019).

### AMD burden by education in 2017

**Fig. 5:**
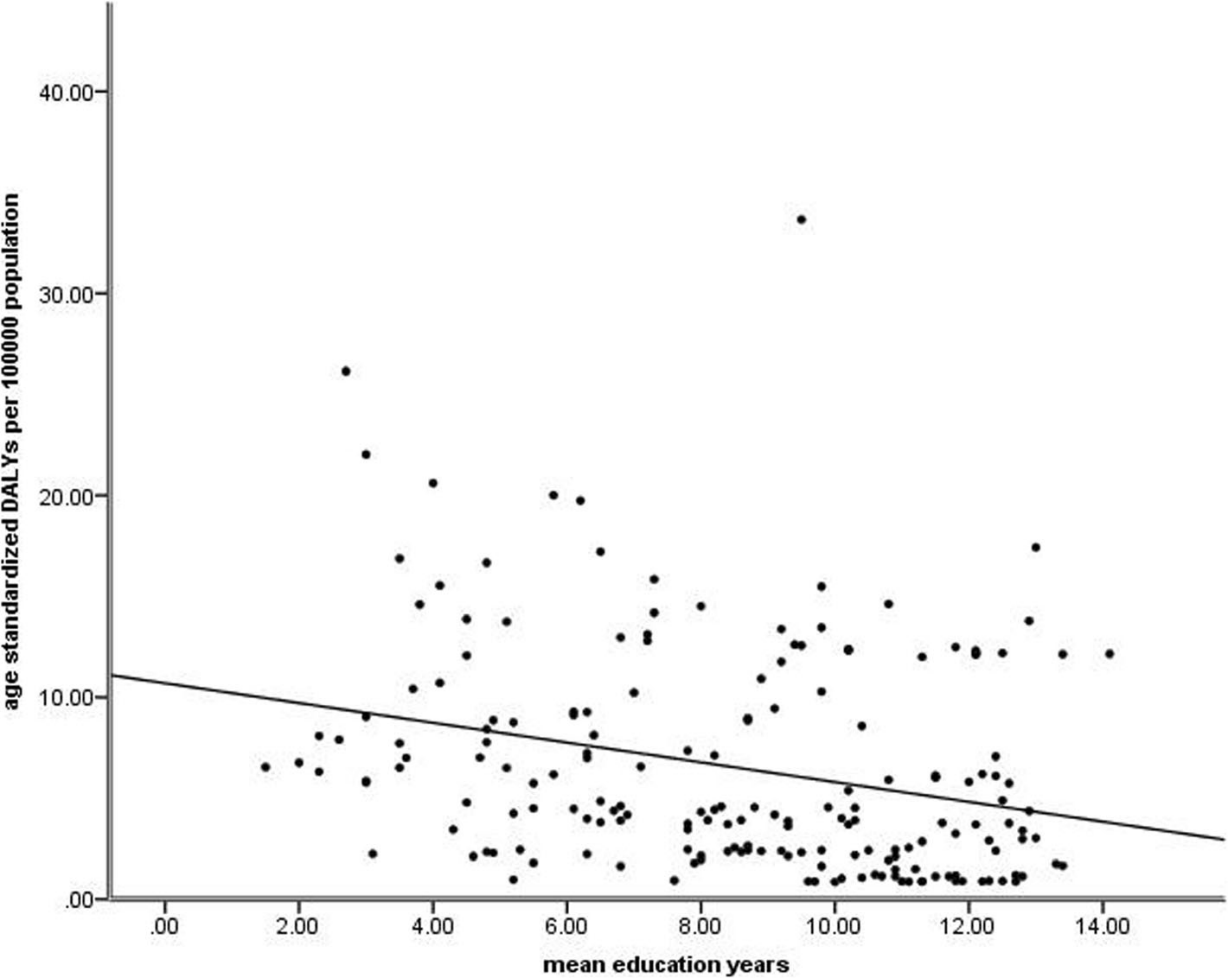
Global burden of Age-related Mcular Degeneration (AMD) in terms of age-standardized disability-adjusted life years (DALY) rates by mean education years in 2017

Linear regression analysis indicated that mean education years had a significant effect on age-standardized DALY rates (adjusted R^2^ = 0.071; *P* < 0.001). As can be seen in Fig. 5, age-standardized DALY rates were inversely associated with mean education years (standardized β = − 0.276, P < 0.001).

### AMD burden by public health expenditure in 2015

**Fig. 6:**
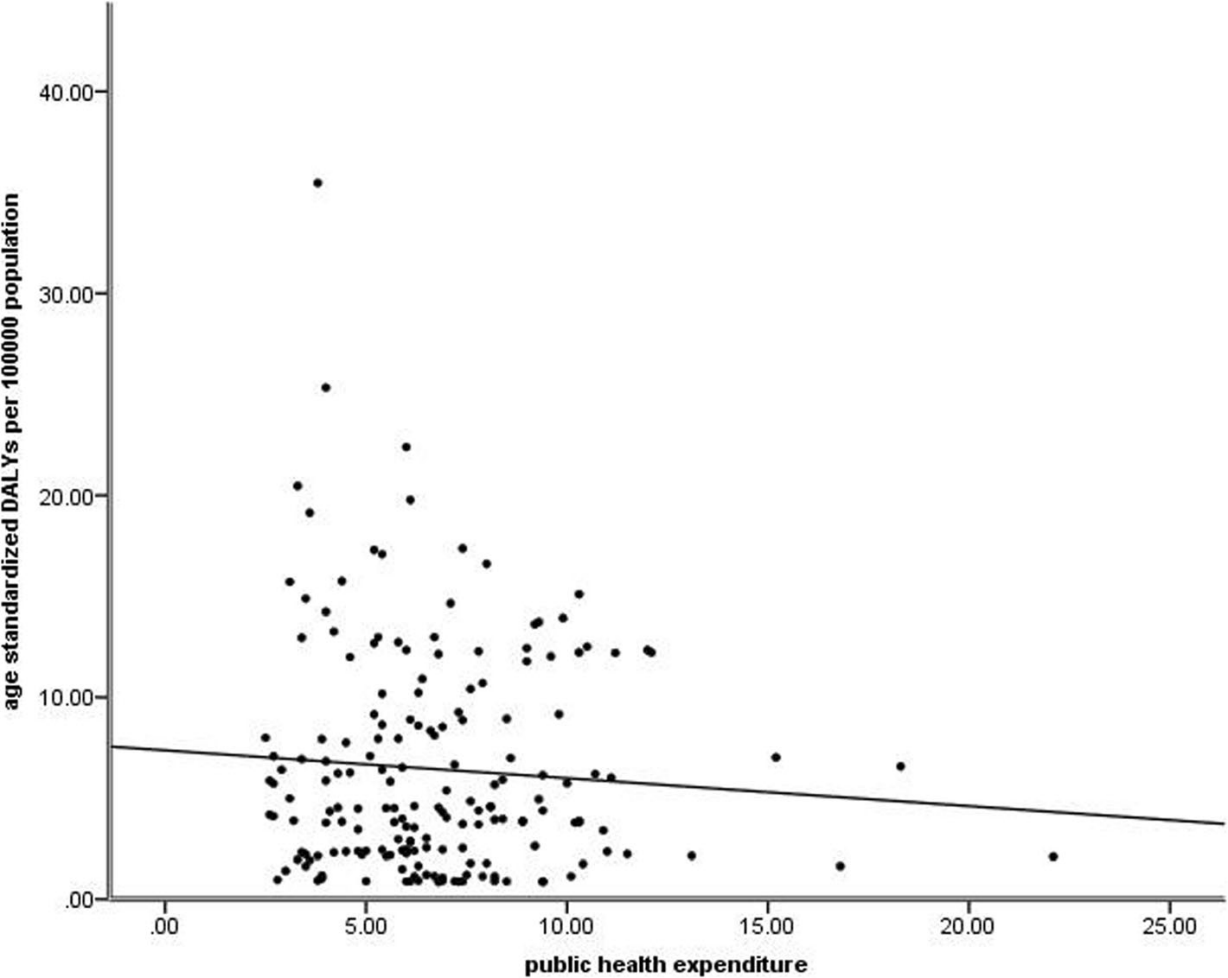
Global burden of Age-related Macular Degeneration (AMD) in terms of age-standardized disability-adjusted life years (DALY) rates by public health expenditure in 2015

Results showed that public health expenditure had no significant effect on age-standardized DALY rates (*P* = 0.331) by linear regression analysis (Fig. 6).

### Construction of a multivariate linear regression model

According to the results above, we included HDI and mean education years as the independent factors to construct a multivariate linear regression model of age standardized DALYs. A significant correlation was found (*P* < 0.001) and this model can explain 11.3% of the variance in regional difference of AMD burden.

## Discussion

This study aimed to explain why different regions held different AMD burden, and made efforts on the roles that latitude, socioeconomic status, education and public health expenditure played in the regional disparity. After excluding the influence of population size and composition, we found that AMD burden decreased recently in terms of age-standardized DALY rates, while the absolute DALY numbers and rates both had an evident increase. This result can be explained as follows: with the development of medical technology and related devices, the diagnostic rates, cure rates and life expectancy increase notably in recent years. On the other hand, the introduction of anti-vascular endothelial growth factor (VEGF) therapy for neovascular AMD has offered remarkable clinical benefits [18], leading to atrophy of neovascularization. Ranibizumab, bevacizumab and aflibercept are the most common drugs used in the therapy of neovascular AMD [19], and the off-label use of bevacizumab is in consideration of reducing the cost [20–22], although some studies also indicated a high frequency of serious adverse events in the patients receiving bevacizumab therapy [23–25]. These all contributed to the recent downtrend of AMD burden.

The patterns of AMD burden in different regions have been controversial so far. Some population studies (the Age-Related Eye Disease Study [26], the Beijing Eye Study [5, 6], the Los Angeles Latino Eye Study [27], and the study [2] by Wong et al.) showed that the prevalence of AMD was higher in the white; still, the statistic results of other studies (the National Health and Nutrition Examination Survey III [28], the multi-ethnic study of atherosclerosis [29], a multiethnic Asian cohort study [7] and the Nagahama study [8]) showed that there were no significant difference among different regions. In our study, results showed that the African and Eastern Mediterranean regions held higher AMD burden than the American, South-East Asian and Western Pacific regions, and the latter three regions shared no significant difference of AMD burden (Fig. 2). Apart from the parts of genetic factors played therein, we cannot deny the roles of environmental factors in the region difference of AMD burden. Therefore, this study was in an attempt to explain this difference from several region-related aspects, such as latitude, socioeconomic status, education level, public health expenditure and so on.

The relationship between latitude and AMD burden has rarely been studied so far. In our study, it was shown that AMD burden was comparable among different latitude groups. In past publications, sun insolation was expected to be related with AMD burden. It was said that high sun insolation destroyed retina pigment epithelium (RPE) cells, which is a critical part in the development of AMD [30]. Besides, the function of vitamin D in the pathogenesis of AMD was also a concern as the immunomodulatory and antiangiogenic properties of vitamin D were assumed to protect persons from AMD [31]. The result in our study did not deny the role of latitude as well as sun insolation in AMD burden, but should be further verified after excluding confounding factors.

**Fig. 7:**
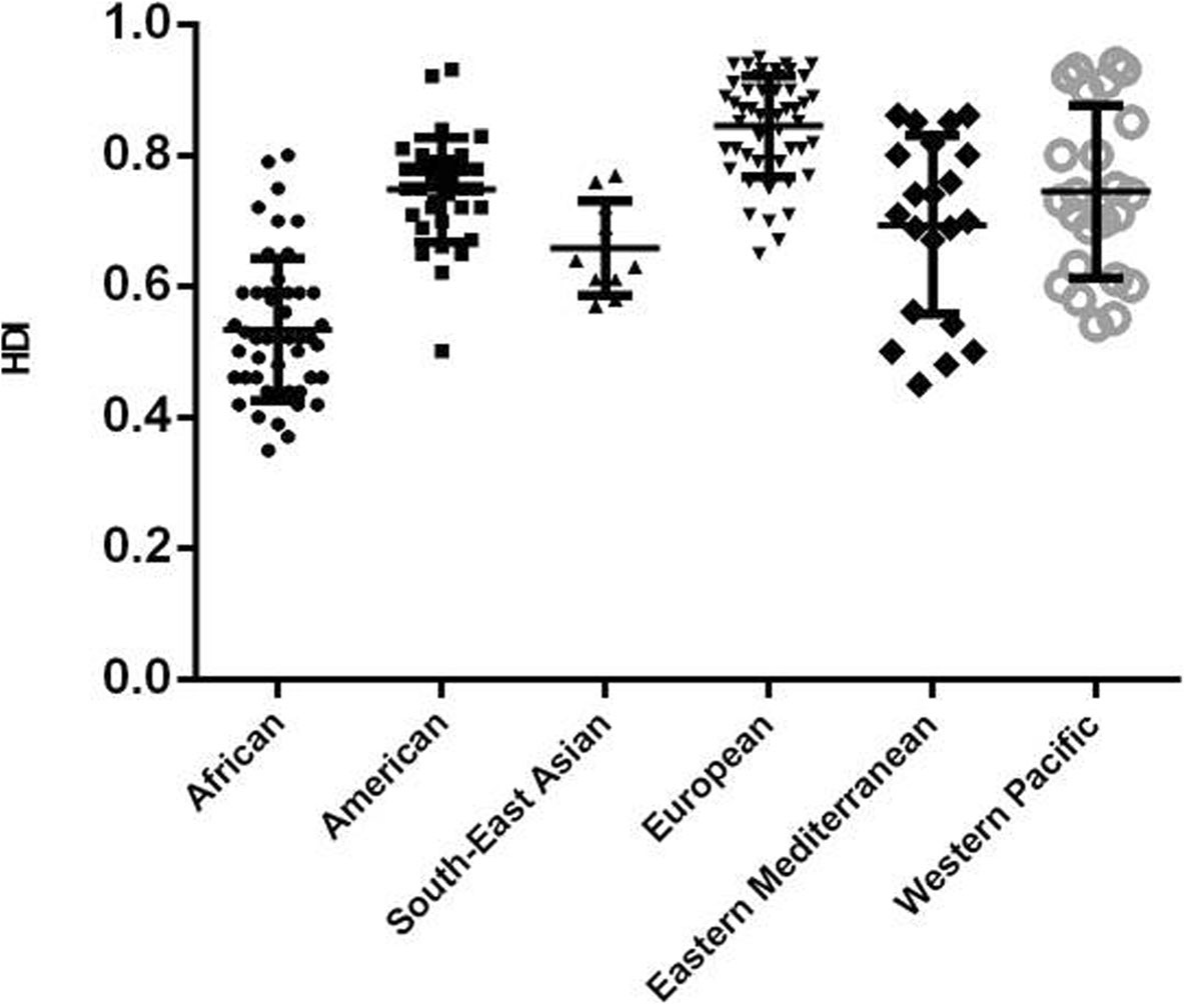
Human Development Index by World Health Organization (WHO) region in 2017. Lines represent 95% uncertainty intervals

It is no wonder that high socioeconomic level contributes to the decrease of disease burden, as more developed countries are expected to be equipped with better healthcare systems and provide more persons with regular physical examinations to realize early detection, diagnosis and treatment of diseases. In our study, AMD burden differed significantly among regions of different socioeconomic status, and a negative relationship existed between these two parameters (Fig. 4). As for WHO region system, HDI differed significantly in different WHO regions (*P* < 0.0001); the countries of the European region, whose AMD burden was low, had the highest HDI; while African countries with high AMD burden held the lowest one, with the rest four regions sharing no significant difference of HDI (Fig. 7).

**Fig. 8:**
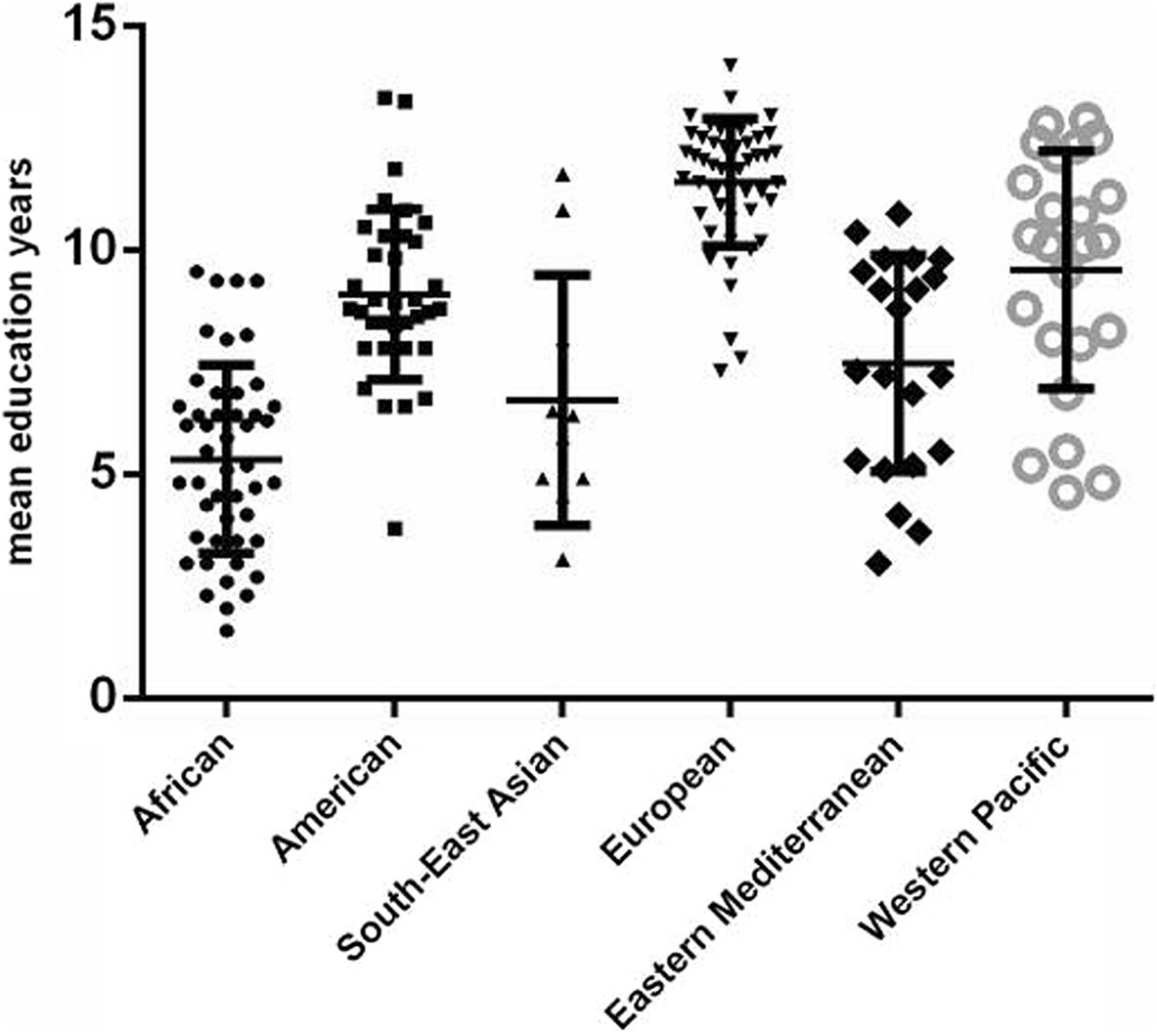
Mean education years of by World Health Organization (WHO) region in 2017. Lines represent 95% uncertainty intervals

To deeply probe the underlying factors that impacted the region disparity of AMD burden, we further analyzed the relationship between education level/Public Health Expenditure and AMD burden. It was not surprising to find that the AMD burden was lighter where mean education years were longer. Low education is related to lack of awareness about health care, thus leading to low visiting rates and low compliance to treatment. Previous studies also showed that individuals with low education were less likely to visit an eye care professional [32–34]. Further interpreting results based on WHO region system, mean education years differed in different WHO regions. Also, the European region provided the most education years, undoubtedly owning a comparatively low AMD burden. By contrast, the African region with a comparatively high AMD burden provided the fewest education years (Fig. 8). The role of public health expenditure in AMD burden was not obvious in this study; however, the lack of data about the discrete public health expenditure on AMD made this result not so convincing, as different governments treated AMD in their own ways according to prevalence and case fatality rate of AMD in the certain country.

DALYs have its own unique computing method different from the universal epidemiological indicators (prevalence, incidence, et al.) [10], which is the total of years lived with disability (YLDs) and years of life lost (YLLs) owing to premature death. So the application of DALYs can provide a novel insight into the global burden of diseases. Owen et al. [35] showed in 2012 that the estimated prevalence of late AMD was 2.4% (95% credible interval (CrI) 1.7 to 3.3%) in UK 2007–2009 population, while ISAO NAKATA et al. [8] represented in the article that the overall prevalence in 2008–2010 Japanese population of early and late AMD were 22.8% (95% confidence interval (CI) 21.7–24.0%) and 0.58% (95% CI 0.36–0.80%) respectively. This result is consistent with past publication [2] that the white own higher prevalence though contradictory to what we found in this study. It is a reasonable speculation that DALYs are highly influenced by socioeconomic status, education level and public health expenditure as listed above, and the white who own advanced socioeconomic development, excellent education environment and high public health expenditure undoubtedly have low DALYs.

This study was subjected to the limitations which had been noted in the GBD 2017 study, such as data sources and statistical assumption [13, 14]. Besides, the division of different latitude groups was based on which latitude range the majority of areas was located on, and the existence of countries covering more than one latitude range might influence the accuracy of results.

## Conclusion

This study is the first to explain the regional differences of AMD burden from latitude, socioeconomic status, education and public health expenditure. The results showed a decreased level of global burden of AMD recently with significant disparity across different WHO regions; the African region with the lowest HDI and the fewest education years had a considerably high AMD burden; and the European region in the contrary with the highest HDI and the most education years owned a fairly low AMD burden. This phenomenon highlights the need to reduce the AMD burden especially in the developing countries, and pay more attention to education with related policy improved and publicity strengthened. More discrete data are needed to support the roles of latitude and public health expenditure in regional differences of AMD burden.

## Data Availability

The datasets generated during and/or analysed during the current study are available in the Global Health Data Exchange repository, http://ghdx.healthdata.org/gbd-results-tool and United Nations Development Programme repository, http://hdr.undp.org/.

## Abbreviations

AMD: Age-related macular degeneration
DALYs: Disability-adjusted life years
GBD: Global burden of disease
HDI: Human development index
RPE: Retina pigment epithelium
VEGF: Vascular endothelial growth factor
WHO: World health organization
YLDs: Years lived with disability
YLLs: Years of life lost

## References

[1] Pennington KL, Deangelis MM (2016). Epidemiology of age-related macular degeneration (AMD): associations with cardiovascular disease phenotypes and lipid factors. Eye Vis.

[2] Wong WL, Su X, Li X, Cheung CM, Klein R, Cheng CY (2014). Global prevalence of age-related macular degeneration and disease burden projection for 2020 and 2040: a systematic review and meta-analysis. Lancet Global Health.

[3] Pascolini D, Mariotti SP (2012). Global estimates of visual impairment: 2010. Br J Ophthalmol.

[4] Prevent Blindness. Cost of Vision Problems. https://www.preventblindness.org/cost-vision-problems. Accessed July 2018.

[5] Li Y, Xu L, Jonas JB, Yang H, Ma Y, Li J (2006). Prevalence of age-related maculopathy in the adult population in China: the Beijing eye study. Am J Ophthalmol.

[6] Li Y, Xu L, Wang YX, You QS, Yang H, Jonas JB (2008). Prevalence of age-related maculopathy in the adult population in China: the Beijing eye study. Am J Ophthalmol.

[7] Cheung CM, Tai ES, Kawasaki R, Tay WT, Lee JL, Hamzah H (2012). Prevalence of and risk factors for age-related macular degeneration in a multiethnic Asian cohort. Arch Ophthalmol.

[8] Nakata I, Yamashiro K, Nakanishi H, Akagi-Kurashige Y, Miyake M, Tsujikawa A (2013). Prevalence and characteristics of age-related macular degeneration in the Japanese population: the Nagahama study. Am J Ophthalmol.

[9] Cheung CM, Li X, Cheng CY, Zheng Y, Mitchell P, Wang JJ (2014). Prevalence, racial variations, and risk factors of age-related macular degeneration in Singaporean Chinese, Indians, and Malays. Ophthalmology.

[10] Global Health Data Exchange Database. GBD Results Tool. http://ghdx.healthdata.org/gbd-results-tool. Accessed July 2019.

[11] Murray CJ, Ezzati M, Flaxman AD, Lim S, Lozano R, Michaud C (2012). GBD 2010: design, definitions, and metrics. Lancet.

[12] GBD 2015 Mortality and Causes of Death Collaborators (2016). Global, regional, and national life expectancy, all-cause mortality, and causespecific mortality for 249 causes of death, 1980–2015: a systematic analysis for the Global Burden of Disease Study 2015. Lancet.

[13] GBD Disease, Incidence I, Collaborators P (2018). Global, regional and national incidence, prevalence, and years lived with disability for 354 diseases and injuries, 1990–2017: a systematic analysis for the Global Burden of Disease Study 2017. Lancet.

[14] GBD DALYs, Collaborators H (2018). Global, regional, and national disability-adjusted life-years (DALYs) for 359 diseases and injuries and healthy life expectancy (HALE) for 195 countries and territories, 1990-2017: a systematic analysis for the global burden of Disease study 2017. Lancet.

[15] United Nations Development Programme Database. Human development report 2018: work for human development. http://hdr.undp.org/. Accessed July 2019.

[16] Nachar N. The Mann-Whitney U: a test for assessing whether two independent samples come from the same distribution. Tutorials Quant Methods Psychol. 2008;4.

[17] Chan Y, Walmsley RP (1997). Learning and understanding the Kruskal-Wallis one-way analysis-of-variance-by-ranks test for differences among three or more independent groups. Phys Ther.

[18] Rothen M, Jablon E, Monares G, Fontal MR, Alfaro DV (2005). Anti-macular degeneration agents. Ophthalmol Clin N Am.

[19] Avery RL, Castellarin AA, Steinle NC, Dhoot DS, Pieramici DJ, See R (2014). Systemic pharmacokinetics following intravitreal injections of ranibizumab, bevacizumab or aflibercept in patients with neovascular AMD. Br J Ophthalmol.

[20] Investigators IS, Chakravarthy U, Harding SP, Rogers CA, Downes SM, Lotery AJ (2012). Ranibizumab versus bevacizumab to treat neovascular age-related macular degeneration: one-year findings from the IVAN randomized trial. Ophthalmology.

[21] Kodjikian L, Souied EH, Mimoun G, Mauget-Faÿsse M, Behar-Cohen F, Decullier E (2013). Ranibizumab versus Bevacizumab for Neovascular age-related macular degeneration: results from the GEFAL noninferiority randomized trial. Ophthalmology.

[22] Krebs I, Schmetterer L, Boltz A, Told R, Vécsei-Marlovits V, Egger S (2013). A randomised double-masked trial comparing the visual outcome after treatment with ranibizumab or bevacizumab in patients with neovascular age-related macular degeneration. Br J Ophthalmol.

[23] Chakravarthy U, Harding SP, Rogers CA, Downes SM, Lotery AJ, Culliford LA (2013). Alternative treatments to inhibit VEGF in age-related choroidal neovascularisation: 2-year findings of the IVAN randomised controlled trial. Lancet.

[24] Martin DF, Maguire MG, Fine SL, Ying GS, Jaffe GJ, Comparison of Age-related Macular Degeneration Treatments Trials (CATT) Research Group (2012). Ranibizumab and Bevacizumab for treatment of Neovascular age-related macular degeneration: 2-year results. Ophthalmology.

[25] Martin DF, Maguire MG, Ying GS, Grunwald JE, Fine SL, CATT Research Group (2011). Ranibizumab and Bevacizumab for Neovascular age-related macular degeneration. N Engl J Med.

[26] Age-Related Eye Disease Study Research Group (2000). Risk factors associated with age-related macular degeneration: a case-control study in the age-related eye disease study: age-related eye disease study report number 3. Ophthalmology.

[27] Fraser-Bell S, Donofrio J, Wu J, Klein R, Azen SP, Varma R (2005). Sociodemographic factors and age-related macular degeneration in Latinos: the Los Angeles Latino eye study. Am J Ophthalmol.

[28] Klein R, Klein BE, Jensen SC, Mares-Perlman JA, Cruickshanks KJ, Palta M (1999). Age-related maculopathy in a multiracial United States population: the National Health and nutrition examination survey III. Ophthalmology.

[29] Klein R, Klein BEK, Knudtson MD, Wong TY, Cotch MF, Liu K (2006). Prevalence of age-related macular degeneration in 4 racial/ethnic groups in the multi-ethnic study of atherosclerosis. Ophthalmology.

[30] Sparrow JR, Boulton M (2005). RPE lipofuscin and its role in retinal pathobiology. Exp Eye Res.

[31] Reibaldi M, Longo A, Pulvirenti A, Avitabile T, Russo A, Cillino S (2016). Geo-epidemiology of age-related macular degeneration: new clues into the pathogenesis. Am J Ophthalmol.

[32] Orr P, Barrón Y, Schein OD, Rubin GS, West SK (1999). Eye care utilization by older Americans: the SEE project. Salisbury Eye Eval Ophthalmol.

[33] Ellwein LB, Urato CJ (2002). Use of eye care and associated charges among the Medicare population: 1991-1998. Arch Ophthalmol.

[34] Bailey RN, Indian RW, Zhang X, Geiss LS, Duenas MR, Saaddine JB (2006). Visual impairment and eye care among older adults-five states, 2005. MMWR Morb Mortal Wkly Rep.

[35] Owen CG, Jarrar Z, Wormald R, Cook DG, Fletcher AE, Rudnicka AR (2012). The estimated prevalence and incidence of late stage age related macular degeneration in the UK. Br J Ophthalmol.

